# Risk factors for cervical HPV infection and genotypes distribution in HIV-infected South Brazilian women

**DOI:** 10.1186/1750-9378-9-6

**Published:** 2014-02-11

**Authors:** Sheila C Rocha-Brischiliari, Fabrícia Gimenes, André L P de Abreu, Mary M T Irie, Raquel P Souza, Rosangela G Santana, Angela A F Gravena, Maria D de B Carvalho, Marcia E L Consolaro, Sandra M Pelloso

**Affiliations:** 1 Department of Nursing, State University of Maringá, Paraná, Brazil; 2 Department of Clinical Analysis and Biomedicine, State University of Maringá, Paraná, Brazil; 3 Department of Statistics, State University of Maringá, Paraná, Brazil; 4 Department of Medicine, State University of Maringá, Paraná, Brazil

**Keywords:** HIV, HPV, Genotypes, Risk factors, Cervical lesions, Cervical cancer

## Abstract

**Background:**

Human *Papillomavirus* (HPV) infection is particularly burdensome for women infected with human immunodeficiency virus (HIV), which increases their risk of developing cervical lesions and cancer (CC). We conducted a molecular study of the distribution of cervical HPV genotypes and the risk factors for this infection in HIV-infected Brazilian women.

**Findings:**

Cervical and endocervical samples for Papanicolaou screening and HPV detection were collected from 178 HIV-infected women using highly active antiretroviral therapy (HAART) of Maringá city/Brazil. Risk factors were assessed using a standardized questionnaire, and the data regarding to HIV infection from medical records. HPV was detected by polymerase chain reaction (PCR), and genotyping using PCR-restriction fragment length polymorphism analysis. HIV infection was well controlled, but women with a current CD4+ T lymphocyte count between 200–350 cells/mm^3^ (37.6%) had a two-fold greater risk of HPV infection than those with > 350 cells/mm^3^ (26.4%). HPV was associated with parity ≥3, hormonal contraceptive use and current smoker. HPV infection occurred with high frequency (46.6%) but a low frequency of cervical abnormalities was detected (7.30%), mainly low-grade squamous intraephitelial cervical lesions (LSIL) (84.6%). A high frequency of multiple HPV infections was detected (23.0%), and the most frequent HPV genotype was HPV-72 (6.7%), followed by −16, -31 and -51 (6.14% each).

**Conclusions:**

We showed that HAART use does not protect HIV-infected women from HPV, but appear to exert some protection against cervical lesions development. This study provides other important information about risk factors and cervical HPV in HIV-infected women, which can contribute to planning protocols.

## Findings

It is estimated that in 2020, cervical cancer (CC) will be diagnosed in over 665,035 women worldwide, and 357,852 will die as a result [1]. The frequency of CC is much higher in underdeveloped or developing countries than it is in developed countries [2]. In Brazil, CC is the third most common cancer among women, with 17.540 new cases diagnosed in 2012 [3].

The association between persistent high-risk (HR) Human *Papillomavirus* (HPV) and CC has been well established [4]. HPV infection is particularly burdensome for human immunodeficiency virus (HIV)-infected women, as they are more vulnerable to infection and are less likely to clear the virus, which increases their risk of developing cervical lesions and cancer. Moreover, in HIV-infected women, CC responds poorly to the recommended therapies, is more aggressive, and in cases of recurrence, has a worse prognosis [5]. In Brazil, approximately 180,000 HIV-positive individuals are undergoing highly active antiretroviral therapy (HAART) administered by the Public Health System [6]. While this therapy has been associated with a substantial reduction in AIDS-related mortality, its role in preventing HPV infection and progression to CC is still poorly studied and controversial [6,7].

Studies have unanimously showed that HIV-infected women are more commonly infected with non-16 and −18 HR-HPV genotypes, such as 52 and 58 [8,9]. Given that current vaccines target HPV -16/-18, these findings may have important implications for future HPV vaccines that target other types of HPV that are associated with disease risk in HIV-infected women [10].

Considering that epidemiological data from different populations are required for public policies addressing CC prevention in HIV-infected women and that few studies from Brazil and Latin America have collected these data, we conducted a molecular study of the distribution of cervical HPV genotypes and the risk factors associated with this infection in HIV-infected Brazilian women.

In total, 178 HIV-infected women using HAART, aged 18 to 66 years, who attended the Specialized Assistance Service (SAE) for sexually transmitted diseases (STD)/AIDS of Maringá city/Southern Brazil, from April 1 to October 30, 2011, were included. The inclusion criterion required that the women had been diagnosed twice with HIV/AIDS using different methods and using HAART. The exclusion criteria were previous hysterectomy, current or recent pregnancy, age younger than 18 years, and no history of sexual activity.

Of the 424 HIV-infected women enrolled in the SAE, 100 were excluded, and a total of 324 were eligible for the study. The sample size was calculated with an HPV prevalence of 50%, confidence interval of 95%, error estimate of 5%. With an increase of 10% for possible participant losses, the total sample size was fixed at 178 randomly selected women.

The women were interviewed using a standardized questionnaire to obtain socio-economic and demographic information, obstetric and gynecologic history and data on their sexual behavior. Data regarding HIV infection were obtained from SAE medical records. A single nursing contacted all of the women, administered the questionnaire and collected the cervical samples. This project was approved by the Committee for Ethics in Research Involving Humans at the State University of Maringá (UEM)/Paraná, Brazil (nº 085/2011).

**Figure 1:**
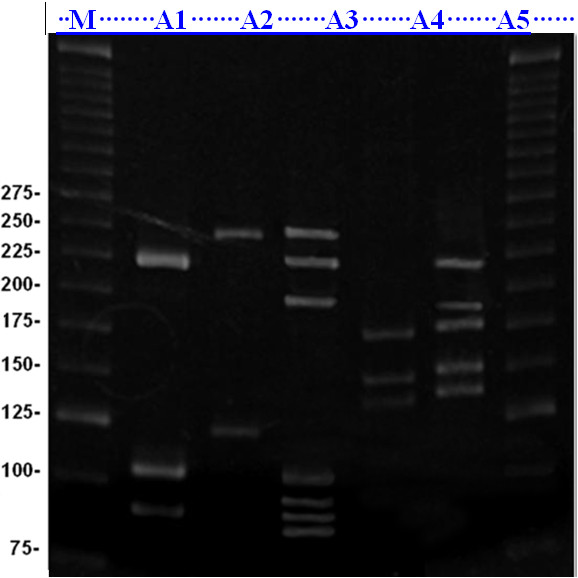
**Electrophoretic analysis of HPV genotyping by PCR-restriction fragment length polymorphism analysis (PCR-RFLP) using *Hpy*CH4V in 8% polyacrylamide gel stained with ethidium bromide.** Sample **A**1, genotype −31 (HR) in single HPV infection (216, 108, 94 base pairs-pb); **A**2, genotype −56 (HR) in single HPV infection (244, 121 pb); **A**3, genotypes −13 (LR), -16 and −58 (HR) in multiple HPV infection (244, 216, 191, 103, 99, 91 e 89 pb); **A**4, genotype −51 (HR) in single HPV infection (171, 147, 137 pb); **A**5, genotypes −16 (HR) and −61 (LR) on double HPV infection (216, 191, 171, 147, 137). M, molecular weight marker (25 base pairs).

Ecto/endocervical samples were collected with an Ayre’s spatula and cytobrush for cervical cytology (Papanicolaou screening) and polymerase chain reaction (PCR); the samples were suspended in 1 ml of 0.9% NaCl solution and stored at -20°C. The cytological smears were sent to the Clinical Cytology Laboratory of UEM and were graded according to the Bethesda System [11]. Genomic DNA was extracted using an AxyPrep™ Body Fluid Viral DNA/RNA Miniprep kit (Axygen, CA, USA). PCR amplification of HPV was carried out using the following primers: MY09 (5’CGTCCMAARGGAWACTGATC-3’)/MY11(5’-GCMCAGGGWCATAAYAATGG-3’). Genotyping by PCR-restriction fragment length polymorphism analysis using *Hpy*CH4V [12] was performed. Co-amplification of the human β-globin gene was performed, as an internal control, using the following primers under the same conditions as those used for HPV-PCR: GH20 (5’-GAAGAGCCAAGGACAGGTAC-3’)/PC04 (5’-CAACTTCATCCACGTTCACC-3′) (Figure 1).

Statistical analysis was performed using Open Source Epidemiologic Statistics for Public Health/OpenEpi, Version 2.3.1. All variables were expressed as absolute and relative frequencies. For univariate analysis, categorical variables were compared with HPV infection by χ^2^-square and Fisher’s exact test. Some variables of interest with p < 0.20 were selected for logistic regression analysis. A crude odds ratio (OR) and 95% confidence interval (CI) were calculated. A p-value < 0.05 was considered significant.

Most HIV-infected women showed excellent control of the HIV infection, based on HAART proper use (79.2%), high current CD4+ T lymphocyte count (37.6% with 200–350 cells/mm^3^ and 26.4% with > 350 cells/mm^3^) and low current viral load (58.4% < minimum limit and 38.8% between the minimum limit and 100 copies/ml).

Univariate analysis revealed an association between HPV infection and parity ≥ 3 (p = 0.01), not undergoing Pap screening in the last 3 years (p = 0.03), current smoking (p = 0.01), previous smoking (p = 0.05) and current CD4+ T lymphocyte count < 200 cells/mm^3^ (p = 0.06) (Table 1).

**Table 1:** Univariate analysis of epidemiologic and HIV infection data in 178 women and association with HPV

**Variables**	**n (%)**	**HPV (%)**	**OR (95% CI)**^**a**^	**p value**^**b**^
**Age ranges (years)**				
18-30	28 (15.7)	60.7	1.93 (0.94-1.99)	0.13
31-40	60 (33.7)	43.3	0.96 (0.47-1.95)	0.89
> 40	90 (50.6)	44.4	1.0	
**Education (years)**				
< 8	85 (47.8)	45.9	1.69 (0.93-3.06)	0.84
≥ 8	93 (52.2)	47.3	1.0	
**Marital status**				
Married	93 (52.2)	45.2	1.0	
Unmarried	31 (17.4)	48.4	1.14 (0.46-2.77)	0.75
Widowed	54 (30.3)	48.1	1.13 (0.55-2.33)	0.72
**Skin color**				
White	114 (64.0)	42.1	1.0	
Not white	64 (36.0)	54.7	1.66 (0.86-3.22)	0.10
**Menarche (years)**				
< 13	95 (53.4)	43.2	0.74 (0.39-1.40)	0.32
≥ 13	83 (46.6)	50.6	1.0	
**Age of sexual debut (years)**				
< 18	112 (62.9)	45.5	0.89 (0.46-1.71)	0.70
≥ 18	66 (37.1)	48.5	1.0	
**Sexual partners (number)**				
1	16 (9.0)	56.2	1.0	
2-7	74 (41.6)	44.6	0.63 (0.18-2.10)	0.39
> 7	88 (49.4)	46.6	0.68 (0.20-2.22)	0.47
**Parity (number)**				
0	20 (11.2)	70.0	1.0	
1-2	67 (37.7)	49.2	0.42 (0.12-1.35)	0.10
≥ 3	91 (51.1)	39.6	0.28 (0.09-0.88)	0.01
**History of Pap screening in the past three years**				
Yes	143 (80.3)	42.7	1.0	
No	35 (19.7)	62.9	2.27 (1.01-5.23)	0.03
**Hormonal contraceptive use**				
Yes	135 (75.9)	43.7	0.61 (0.29-1.30)	0.16
No	43 (24.1)	55.8	1.0	
**Gynecologic infections**				
Yes	127 (71.3)	48.0	1.22 (0.60-2.47)	0.55
No	51 (28.7)	43.1	1.0	
**Gynecologic infections (number)**				
0	55 (30.9)	40.0	1.0	
1-2	93 (52.2)	48.4	1.41 (0.68-2.92)	0.32
≥ 3	30 (16.9)	53.3	1.71 (0.64-4.64)	0.23
**Cigarette smoking**				
Yes	35 (19.7)	65.7	2.76 (1.15-6.67)	0.01
No	100 (56.2)	41.0	1.0	
Ex-smoker	43 (24.1)	44.2	0.41 (0.15-1.14)	0.05
**Period of HIV infection (years)**				
< 5	76 (42.7)	56.6	1.72 (0.79-3.75)	0.13
5-10	51 (28.6)	35.5	0.72 (0.30-1.72)	0.41
> 10	51 (28.6)	43.1	1.0	
**HAART correct use**				
Yes	141 (79.2)	46.8	1.04 (0.47-2.28)	0.92
No	37 (20.8)	45.9	1.0	
**Current CD4+ T lymphocyte (cells/mm**^**3**^**)**				
< 200	15 (8.4)	66.7	2.76 (0.81-9.92)	0.06
200-350	67 (37.6)	56.3	1.78 (0.76-4.16)	0.14
> 350	47 (26.4)	42.0	1.0	
**CD4+ T lymphocyte range (cells/mm**^**3**^**)**				
< 200	64 (35.9)	51.5	1.21 (0.53-2.76)	0.62
200-350	67 (37.6)	41.8	0.82 (0.36-1.85)	0.59
> 350	47 (26.4)	46.8	1.0	
**Recent viral load (copies/ml)**				
< L min	104 (58.4)	43.3	1.0	
1-100.000	69 (38.8)	49.3	1.27 (0.66-2.46)	0.43
> 100.000	5 (2.8)	80.0	5.24 (0.52-27.6)	0.17
**Viral load range (copies/ml)**				
1-100.000	50 (28.1)	50.0	1.0	
> 100.000	128 (71.9)	45.3	0.83 (0.41-1.68)	0.57

^a^Odds ratio (*OR*) 95% confidence interval (*CI*).

^b^A p-value < 0.05 was considered significant.

Highly active antiretroviral therapy (*HAART*).

Papanicolaou secreening (Pap screening).

After logistic regression, HPV infection was associated with parity ≥ 3 (p < 0.01; OR = 0.17, 0.05-0.55), hormonal contraceptive use (p = 0.03; OR = 0.40, 95% CI = 0.17-0.92), current smoking (p = 0.01; OR = 95% CI = 3.04, 0.24-7.46), and current CD4+ T lymphocyte count < 200 cells/mm^3^ (p = 0.08; OR = 3.04, 95% CI = 0.86-10.6 ) and between 200–350 cells/mm^3^ (p = 0.02; OR = 1.66, 95% CI = 1.05-2.62 ) (Table 2).

**Table 2:** Multivariate analysis of epidemiologic and HIV infection data in 178 women and association with HPV

**Variables**	**Adjusted OR**	**95% CI**^**a**^	**p value**^**b**^
**Skin color**	1.46	0.71-3.02	0.30
**Parity number = 0**	1.45	0.14-1.45	0.18
**Parity number ≥ 3**	0.17	0.05-0.55	< 0.01
**History of Pap smear in the past three years**	2.08	0.86-5.00	0.10
**Hormonal contraceptives use**	0.40	0.17-0.92	0.03
**Smoker**	3.04	1.24-7.46	0.01
**Ex-smoker**	1.42	0.62-3.24	0.40
**HIV infection for 5 years or less**	1.93	0.85-4.35	0.11
**HIV infection between 5–10 years**	1.51	0.20-129	0.15
**CD4+ T lymphocyte < 200 (cells/mm**^**3**^**)**	3.04	0.86-10.6	0.08
**CD4+ T lymphocyte < 200–350 (cells/mm**^**3**^**)**	1.66	1.05-2.62	0.02

^a^Odds ratio (*OR*) 95% confidence interval (*CI*).

^b^A p-value < 0.05 was considered significant.

Papanicolaou secreening (Pap screening).

Cells per cubic millimeter (cells/mm^3^).

A total of 7.3% (n = 13) of the women showed cervical abnormalities, of which 84.6% (n = 11) were low-grade squamous intraepithelial cervical lesions (LSIL) and 7.7% (n = 1) for both atypical squamous cervical cells (ASC) of undetermined significance (ASC-US) and ASC could not be excluded a high-grade squamous intraepithelial cervical lesion (ASC-H) (Table 3).

**Table 3:** Single and multiple HPV infections in 178 women with HIV

**Pap screening findings**	**Single HPV infections**						**Multiple HPV infections**					
	**LR-HPV**		**HR-HPV**		**Total**		*LR-HPV		**HR-HPV		**Total**	
	**n**	**%**	**n**	**%**	**n**	**%**	**n**	**%**	**n**	**%**	**n**	**%**
NILM (n = 165)	14	8.5	23	13.4	^#^37	88.1	7	4.2	^###^26	15.8	^#^33	20.0
ASC-US (n = 1)	-	-	-	-	-	-	-	-	1	100	1	100
ASC-H	-	-	-	-	-	-	1	100	-	-	1	100
(n = 1)												
LSIL (n = 11)	2	18.2	3	27.3	5	11.9	2	18.2	^###^4	36.4	6	54.6
Total (n = 178)	^##^16	9.0	^##^26	14.6	42	100.0	^##^10	5.6	^##^31	17.4	41	23.0

Pap (Papanicolaou) screening with normal cervical cells (NILM).

Atypical squamous cervical cells of undetermined significance (*ASC-US*).

Atypical squamous cervical cells could not be excluded a high-grade squamous intraepithelial cervical lesion (*ASC-H*).

Low-grade squamous intraepithelial cervical lesions (*LSIL*).

*Multiple HPV infections without high- risk HPV (HR-HPV) involvement (i.e. low-risk-*LR* and/or undetermined-risk HPV).

**Multiple HPV infections with HR-HPV involvement (i.e. HR and/or undetermined-risk and/or LR-HPV).

^#^HPV infection was more prevalent in women with normal cytology (NILM) (p = 0.022).

^##^LR- and HR-HPV were detected with similar prevalence in single or multiple HPV infections (p = 0.0727 and 0.0949, respectively).

^###^In multiple HPV infections, HR-HPV was more prevalent than LR in women with NILM and LSIL (p *=* 0.01).

The frequency of HPV was 46.6% (n = 83), and in 23.0% of participants (n = 41) multiple HPV genotypes were detected. Low-risk (LR)-HPV was detected in 14.6% (n = 26), and HR-HPV was detected in 32.0% (n = 57) of the women (p < 0.01). LR and HR-HPV showed similar frequencies in single and multiple infections (p = 0.0727 and 0.0949, respectively). However, in multiple HPV infections, HR-HPV was more frequent than LR-HPV in both women with normal cervical cells (NILM) (n = 26, 15.8% and n = 7, 4.2%, respectively) and those with LSIL (n = 31, 17.4% and n = 10, 5.6%, respectively) (p = 0.01). HPV infection occurred most commonly in women with NILM (n = 72, 40.4%) (p = 0.022) (Table 3).

A total of 37 different HPV genotypes were detected in the 178 women studied. The most frequent HR-HPV genotypes were HPV 16, 31 and 51 (6.18% each); HPV 66 and 58 (3.4% each); HPV 59 and 82 (2.8% each); and HPV 56 and 69 (2.3% each). The most commonly detected LR-HPV genotypes were HPV 72 (6.74%); HPV 61 (3.9%); and HPV 11, 70, 74 and 83 (2.3% each) (Table 4).

**Table 4:** Distribution of 37 HPV genotypes detected in 178 women with HIV

**HPV genotypes**	**Women detected**	
	**n**	**%**
**High-risk (HR)**		
16	11	7.9
31	11	7.9
51	11	7.9
58	6	4.3
66	6	4.3
82	5	3.6
59	5	3.6
18	4	2.9
56	4	2.9
33	3	2.2
73	3	2.2
53	3	2.2
39	1	0.7
45	1	0.7
68	1	0.7
26	1	0.7
**Low-risk (LR)**		
72	12	8.6
61	7	5.1
70	4	2.9
74	4	2.9
83	4	2.9
11	4	2.9
13	3	2.2
62	3	2.2
81	3	2.2
84	3	2.2
55	2	1.4
67	2	1.4
43	2	1.4
6	2	1.4
30	2	1.4
54	1	0.7
44	1	0.7
91	1	0.7
42	1	0.7
69	1	0.7
64	1	0.7

In the present study, we found that although HIV infection was well controlled, women with a current CD4+ T lymphocyte count between 200–350 cells/mm^3^ (37.6%) had a two-fold greater risk of HPV infection than those with > 350 cells/mm^3^ (26.4%). However, our results also showed that although the frequency of HPV was high (46.6%), a low frequency of cervical abnormalities was detected (7.30%), mainly LSIL (84.6%). Recently, it was reported that HPV frequency was much lower (6.7%) in HIV-uninfected women in the city in which this study was conducted [13]. Therefore, HAART use or/and the comprehensive care delivered to these patients through the SAE appears to exert some protection against cervical lesions development in this population.

HPV infection was positively associated with parity number ≥ 3, hormonal contraceptive use and smoker. A relationship between these risk factors and CC has been reported in HIV-uninfected women [14,15]. Our results suggest that they are also associated with HPV infection in HIV-infected women. These data are important for public policies targeting HIV-infected women to prevent HPV infection and CC.

Multiple HPV infections were frequently detected, occurring in half of the HPV positive women; similar findings have already been widely reported [5,10,14]. Interestingly, the most common HPV genotype was −72 (LR, 8.6%), followed by HPV −16, -31 and −51 (7.9% each). These data differ from those described by others in that HR-HPV was detected more frequently. Nonetheless, they are in part agreement with studies showing that these women are commonly infected with the non-18/-16 HR-HPV genotypes [8,9].

In conclusion, we acknowledge that we did not include HIV-uninfected women for comparison. However, we have provided very important information about the risk factors associated with HPV frequency and genotypes in HIV-infected women, and this information can contribute to planning protocols for CC prevention in these patients. It should be noted that the STD/AIDS program of the Brazilian Ministry of Health distributes HAART free of charge and ensures its use. This program also provides comprehensive care to HIV-positive patients, which certainly contributed to the low frequency of cervical lesions (most were LSIL) we observed. This study can serve as a model for the populations of other Latin American countries.

## Abbreviations

HPV: Human papillomavirus; HIV: Human immunodeficiency virus; HR-HPV: High-risk HPV; LR-HPV: Low risk HPV; CC: Cervical cancer; SAE: Specialized Assistance Service; STD: Sexually transmitted disease; AIDS: Acquired immunodeficiency syndrome; PCR: Polymerase chain reaction; LSIL: Low-grade squamous intraepithelial cervical lesions; ASC-UUS: Atypical squamous cervical cells of undetermined significance; ASC-H: Atypical squamous cervical cells could not be excluded a high-grade squamous intraepithelial cervical lesion; OpenEpi: Open source Epidemiologic Statistics for Public Health; OR: Odds ratio; CI: Confidence interval; HAART: Highly active antiretroviral therapy.

## Competing interests

The authors declare that they have no competing interests.

## Authors’ contributions

All of the authors contributed to the manuscript. SCR-B, RPS, FG, and ALPA searched the literature and prepared the manuscript. SCR-B, MDBC and SMP collected the biological samples from the women. MELC, ALPA and FG wrote the manuscript. MELC, RPS, FG and ALPA participated in methodology design and execution. MMTI performed the experiments related to cervical cytology. RGS and AAFG performed the statistical analysis. SCR-B, MDBC and SMP contributed to the statistical analysis and design of the study. MDBC, SMP and MELC were involved in revising the manuscript critically for important intellectual content. MELC revised the final version of the manuscript and provided information and suggestions. All of the authors read and approved the final draft of the manuscript.
